# Adherence to COVID-19 preventive measures among male medical students, Egypt

**DOI:** 10.1186/s42506-022-00103-7

**Published:** 2022-02-17

**Authors:** Haytham Mahmoud Ahmed

**Affiliations:** Department of Public Health and Community Medicine, Faculty of Medicine, Al-Azhar University, Cairo, Egypt

**Keywords:** Adherence, COVID-19 preventive measures, Egypt, Male medical students

## Abstract

**Background:**

Adherence to COVID-19 preventive measures is essential for disease control especially given the lack of effective treatment at the time of data collection. Medical students’ adherence to COVID-19 preventive measures is highly important because they are at a higher risk of infection as they spend considerable time at hospitals for training. In addition, they will be responsible for disease fighting in the future. This study aimed to identify the adherence to COVID-19 preventive measures among the studied male students of Al-Azhar Faculty of Medicine, Egypt.

**Methods:**

An online survey was conducted on a convenience sample of 537 male medical students of Al-Azhar University at the Cairo branch between March 2 and April 15, 2021, using an Arabic questionnaire constructed by Google form with link sharing to students’ groups on WhatsApp and face book applications.

**Results:**

A total of 537 students voluntarily submitted their responses. Most of these students were aged between 18 and 20 years (62.4%), rural residents (62.9%), having family income of 5000 LE or less/month (64.2%), studying at the first, second, and sixth years (79.2%). The prevalence of adherence to COVID-19 preventive measures among studied students was 28.1% without significant difference in the students’ adherence status regarding age, residence, family income, studying year, or academic score. Wearing a face mask outside the house was practiced by 58% of students, avoiding hugging or kissing others (41.3%), and keeping a distance from others (20.7%).

**Conclusion:**

Students showed a low level of adherence to COVID-19 preventive measures among students. Also, there is no significant difference between students’ adherence status in relation to their socio-economic or academic variables. This unhealthy behavior increases the risk of infection among students. Also, students become a source of infection for their vulnerable contacts. More studies are needed to identify the real cause of this low level of adherence.

## Introduction

In December 2019, a novel coronavirus outbreak emerged at Wuhan city, China, resulting in the death of about 1800 and the infection of more than 70,000 in the first 7 weeks of the epidemic. The virus responsible for this outbreak was found to be of the B group coronaviruses. It was named SARS-CoV-2, and the disease was named COVID-19 [1–3].

After about 1 month of the emergence of the disease and on January 30, 2020 (with the rapid spread of the virus around the world), the World Health Organization (WHO) announced the COVID-19 outbreak as a public health emergency of international concern. Countries were asked to apply urgent and aggressive measures to prevent the viral spread. On March 11, 2020, the WHO declared COVID-19 a global pandemic [4].

The rapid spread of the virus around the world was due to its highly contagious nature which distinguishes it from other viruses of the corona family [5]. Also, it was found that the virus is able to survive in the external environment and at room temperature, which doubles the chances of transmission to other people by coming into contact with surfaces contaminated with the virus [6]. Moreover, it has been found that an infected person is able to transmit the infection to 3 people who are contacts, but this number decreases if precautionary measures are taken to prevent infection. The highly infectious nature of the virus is due to the presence of the virus in the respiratory secretions of the infected person in large quantities and for a long period even before the symptoms appear [7]. The US Centers for Disease Control and Prevention (CDC) has recognized that SARS-CoV-2, the virus that causes COVID-19, can be transmitted via respiratory fluids, which are fine droplets released during respiration [8].

The first confirmed case in Egypt was reported in February 14, 2020, at Cairo airport for a Chinese citizen who was also the first confirmed case in Africa [9]. In late February and Early March 2020, multiple cases of the acute respiratory syndrome due to corona virus were discovered in various countries where the patients had a traveling history to Egypt [10]. By March 2, Egypt reported first death from COVID-19 [11].

Egypt’s response was in the form of the closure of airports which went into effect on March 19, 2020, and closure of all schools, universities, and Mosques, along with the cancellation of cultural events and tourist trips. Also, a citizen curfew was imposed as well as forcing the closure of cafes, restaurants, and public places [12, 13]. In parallel to these measures, Egypt started an extensive health education campaign using newspapers, radio, T.V, and social media to increase public knowledge and awareness about COVID-19 [14].

The UN defines youth as people between the ages of 15 and 24 years, and their role has become more important than ever in the efforts to stop the spread of the virus and mitigate its different consequences. For Egypt, a country where the youth constitute about 19% of the population, finding ways to engage young people and empower them can be decisive in the battle against the pandemic [15]. Medical students as future physicians are a subset of the youth who may have more interest and knowledge about COVID-19 than other youth and consequently are supposed to be more adherent to preventive measures than other youth [16]. Meanwhile, they are at a greater risk of infection due to their long stays at the hospitals and their close contact with diseased persons during their study. Moreover, they are mostly not sufficiently trained to protect themselves against infection [17]. This study was conducted to identify male students’ adherence to COVID-19 preventive measures and its predictors.

## Methods

### Study setting and design

This is an online faculty-based survey that was conducted during the academic year 2020–2021 from March 2 to April 15, 2021, to identify the level of adherence to COVID-19 preventive measures and its predictors among students at Al-Azhar Faculty of Medicine for males, Cairo branch. The selection of only male students for this study was due to their accessibility to the authors.

### Sample size and sampling technique

The minimum sample size necessary to perform the study was 357. This number was calculated based on a 95% confidence level, 5% marginal errors, and an adherence proportion of 49.2% [18]. However, to increase the power of the study, no maximum number of participants was decided, and the link remained open throughout the specified period of data collection. In the end, 539 students responded to the questionnaire, of them, two were excluded for not meeting the inclusion criteria of the study (one was female and the other was from the Assiut branch). So, 537 responses represent the sample size of the study.

Non-probability convenience sampling technique (voluntary responses) was used in this study. The questionnaire link was shared to students’ groups on social media (Facebook and WhatsApp) by students’ representatives who were contacted via modules moderators and personal communication. Any male student at the Cairo branch of Al-Azhar Faculty of Medicine who had internet access, WhatsApp/Facebook application, and was willing to participate in the study was included.

### Study tool

Data were collected using a structured self-reported Arabic questionnaire that was constructed based on reviewing the previously published literature [16, 18–21]. The questionnaire was formulated first in English and then translated to Arabic. The electronic form of the questionnaire was constructed using Google forms. The questionnaire was tested for internal consistency and reliability using Cronbach’s *α* test and it was 0.88. A pilot study was performed to test the applicability of the questionnaire and the effectiveness of the online method for data collection. This was done by sending the questionnaire online to 30 students who were contacted personally by the researcher.

The questionnaire link was posted to students’ groups on WhatsApp/Facebook applications by students’ representatives at each studying year. The questionnaire included three sections: the first section included socio-demographic data: age, residence, and monthly family income; and academic data: study year and academic score in the previous year (with exception to the first-year students). The second section included data about COVID-19 infection among students and COVID-19 occurrence and mortality among their relatives and friends. The third section included 15 questions that assessed students’ adherence to COVID-19 preventive measures regarding personal protection (e.g., frequent hand washing, antiseptic use, avoiding the touching of eyes, nose and mouth with hands, and mask wearing), self-isolation, and social distancing. The first thirteen questions used a three-point Likert scale (yes=2, sometimes=1, no=0) while the last two questions were categorized as “yes” or “no” with reversal scoring (yes=0, no=2). The total practice score had a range from 0 to 30. Participants with a total score of 21 points (70% of the total score) or more were considered adherent to COVID-19 preventive measures while those with a score of 20 or less were considered non-adherent to COVID-19 preventive measures [19].

### Ethical approval

All procedures applied in the study comply with the Institutional Research Ethics and the declaration of Helsinki. Approval of the ethics committee at Al-Azhar Faculty of Medicine was obtained. Participation in the study was completely voluntary, and the anonymity of the participants was guaranteed. At the beginning of the questionnaire, there was a written informed consent that must be approved by students to be able to complete the questionnaire. Confidentiality of the collected data was strictly maintained.

### Statistical analysis

Data analysis was performed using Microsoft Excel and SPSS version 20. The extracted Microsoft Excel data file was subjected to editing, sorting, and recoding, and then, the Excel file was imported to the SPSS software. Descriptive statistics (frequencies and percentages) were performed. The differences between the studied variables were analyzed using chi-square tests for qualitative variables. *P* value < 0.05 was considered a sign of significance.

## Results

The highest percent of studied students (45.6%) were aged between 18 and <20 years, were rural residents (62.9%), having family income of 5000 L.E. or less/month (64.2%), studying at the first, second, and sixth years (41.3%, 21.0%, and 16.9% respectively), and have achieved an ʻʻExcellentʼʼ academic score in the previous year (40.6%) (Table 1).

**Table 1:** Socio-demographic characteristics of male students at Al-Azhar Faculty of Medicine, Cairo, Egypt, 2021

Variable	No. (total = 537)	%
Age		
- 18–<20	245	45.6
- 20–<23	180	33.5
- ≥23	112	20.9
Residence		
- Urban	199	37.1
- Rural	338	62.9
Family income		
- Ten thousands L.E. or more/month	44	8.2
- More than 5000 L.E. and less than 10000/month	148	27.6
- Five thousands or less L.E./month	345	64.2
Academic year		
- First	222	41.3
- Second	113	21.0
- Third	50	9.4
- Fourth	42	7.9
- Fifth	16	3.5
- Sixth	91	16.9
Academic score in the previous year (No.= 315) ^a^		
- Excellent	128	40.6
- Very good	94	29.8
- Good	76	24.2
- Accepted	15	4.8
- Failed	2	0.6

^a^First year students were excluded

There were 101 students who had been infected with COVID-19 during the previous year constituting a cumulative incidence of 18.8%. Also, 361 students (67.2%) had relatives and friends who were infected with COVID-19, and 176 students (32.8%) had relatives and friends who died of COVID-19. The latter proportion represented the frequency of deaths among the sample’s relatives and friends in general but not the death rate among the infected ones (Table 2).

**Table 2:** COVID-19 infections among male students at Al-Azhar Faculty of Medicine, Cairo, Egypt, 2021, and their families’ or relatives’ infection or deaths

Variable	No. (total=537)	%
Have you had corona (COVID-19) infection before?		
- Yes	101	18.8
- No	436	81.2
Has any of your relatives or friends been infected with corona (COVID-19)?		
- Yes	361	67.2
- No	176	32.8
Did any of your relatives or friends die of corona (COVID-19)?		
- Yes	176	32.8
- No	361	67.2

**Fig. 1:**
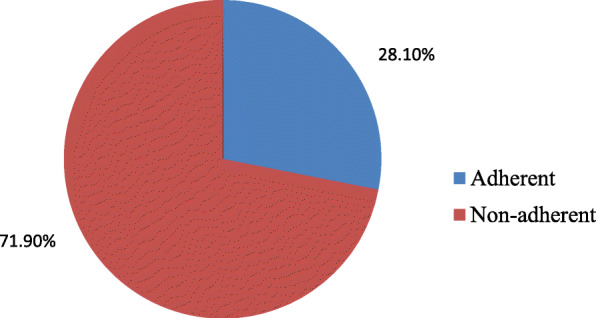
Adherence to COVID-19 preventive measures among male students of Al-Azhar Faculty of Medicine

There were 151 students (representing 28.1% of the total sample) adherent to COVID-19 preventive measures while the remaining 386 students (representing 71.9% of the total sample) were non-adherent to COVID-19 preventive measures (Fig. 1).

Covering the mouth and nose with a tissue (or the elbow) while coughing or sneezing was practiced by 65% of students and 58% wore a face mask when they were outside the house. Also, 41.3% of students avoided touching their eyes, nose, and mouth directly by the finger or the hand and avoided hugging or kissing others whereas 20.7% kept a distance from others and 17.3% avoided shaking hands with others. Moreover, only 25% of students washed their hands frequently with soap and water for at least 20 s, 29.4% used antiseptics, and 22% attended a relative’s or friend’s event in the 2 weeks prior to the survey (Table 3).

**Table 3:** Adhrence to COVID-19 preventive measures among male students at Al-Azhar Faculty of Medicine, Cairo, Egypt, 2021

Adherence item	Yes		Sometimes		No	
	No.	%	No.	%	No.	%
Are you washing your hands frequently with soap and water for 20 s at least?	139	25.9	237	44.1	161	30.0
Are you using antiseptics?	158	29.4	231	43.0	148	27.6
Are you avoiding the eyes, nose, and mouth touching with your fingers or hands?	222	41.3	174	32.4	141	26.3
Are you covering your mouth and nose with a tissue (or elbow) while coughing or sneezing?	349	65.0	118	22	70	13
Are you wearing a face mask outside the house?	316	58.9	173	32.2	48	8.9
Are you disinfecting surfaces and objects in places where you are present?	90	16.8	202	37.6	245	45.6
Are you avoiding crowded places (like malls and markets)?	201	37.4	173	32.2	163	30.4
Are you avoiding hands shaking with others?	93	17.3	242	45.1	202	37.6
Are you avoiding people hugging or kissing?	222	41.3	185	34.5	130	24.2
Are you keeping a distance between you and others (at least 1 m)?	111	20.7	193	35.9	233	43.4
Are you avoiding relatives’ meeting?	69	17.9	145	27.0	296	55.1
Are you avoiding friends’ meetings?	66	12.3	128	23.8	343	63.9
Are you staying at home and do not leave except for necessity?	183	34.1	132	24.6	222	41.3
During the past two weeks: Have you attended any family event (such as marriage, for example)? ^R^	120	22.3	-	-	417	77.7
During the past two weeks: Have you attended any event for your friends (such as marriage, for example)?^R^	119	22.2	-	-	418	77.8

^R^Reversed statement

There is no significant difference in the students’ adherence to COVID-19 in relation to their socio-demographic characters (age, residence, and family income) or academic characters (academic year or academic score) (Table 4).

**Table 4:** Adherence status in relation to socio-demographic and academic characteristics of male students at Al-Azhar Faculty of Medicine, Cairo, Egypt, 2021

Variable	Adherent (total=151)		Non-adherent (total=386)		*P* value
	No.	%	No.	%	
Age					
- 18–<20	69	45.7	176	45.6	
- 20–<23	49	32.5	131	33.9	0.92
- ≥23	33	21.8	79	20.5	
Residence					
- Urban	64	42.4	135	35.0	0.11
- Rural	87	57.6	251	65.0	
Family income					
- Ten thousands LE or more/month	15	9.9	29	7.5	
- More than 5000 LE and less than 10000/month	40	26.5	108	28.0	0.65
- Five thousands or less LE/month	96	63.6	249	64.5	
Academic year					
- First	63	41.6	159	41.2	
- Second	28	18.4	85	22.0	
- Third	16	11.0	34	8.8	0.95
- Fourth	12	7.9	30	7.8	
- Fifth	5	3.2	14	3.6	
- Sixth	27	17.9	64	16.6	
Academic score in the previous year (No.= 315) ^a^					
- Excellent	34	38.6	94	41.6	
- Very good	22	25.0	72	31.8	
- Good	26	29.6	50	22.2	0.4
- Accepted	6	6.8	9	4.0	
- Failed	0	0.0	1	0.4	

Chi^2^ test

^a^First year students are excluded

There is no significant difference in the adherence status of students in relation to their previous infection with COVID-19 or having a relative or friend been infected with COVID-19. However, there is a significant association between the adherence status of students and the deaths of their relatives or friends (Table 5).

**Table 5:** Infections and deaths of COVID-19 and the adherence status of male students at Al-Azhar Faculty of Medicine, Cairo, Egypt, 2021

Variable	Adherent (total=151)		Non-adherent (total=386)		*P* value
	No.	%	No.	%	
Have you had corona (COVID-19) infection before?					
- Yes	27	17.9	74	19.2	0.73
- No	124	82.1	312	80.8	
Had any of your relatives or friends been infected with corona (COVID-19)?					
- Yes	94	62.3	267	69.2	0.13
- No	57	37.7	119	30.8	
Did any of your relatives or friends die of corona (COVID-19)?					
- Yes	31	20.5	145	37.6	0.000^a^
- No	120	79.5	241	62.4	

Chi^2^ test

^a^Statistically significant difference

## Discussion

This study revealed that only 28.1% of the medical students were adherent to COVID-19 preventive measures. Also, when we look closely at the items of adherence, we find that there was a considerable percentage of students who did not always comply with personal protection measures as most of them did not comply with the social distancing and self-isolation measures. This low prevalence of adherence to COVID-19 preventive measures among students despite the expectation based on the fact that they have sufficient knowledge as a result of clinical training and public health study [16] and the relatively long time that elapsed ever since the emergence of the disease along with the extensive health education campaigns may be attributed to the students’ low-risk perceptions where they believe that they are not susceptible to infection with COVID-19 at this age (perceived susceptibility), and even if the infection occurs, there is a low probability of having severe clinical manifestation (perceived severity); and therefore, there is no need for strict adherence to COVID-19 preventive measures. This assumption is supported by the finding of Lee et al. (2021) in Korea where personal beliefs had a significant and robust effect on the practice of the participants [22]. Moreover, a significant positive correlation was found between students’ attitudes and behavior [16]. However, Moradzadeh et al.’s study (2020) in Iran found a significant positive correlation only between the educational level and knowledge but not with the attitude or practice. The authors concluded that improving people’s practice towards COVID-19 preventive measures can be achieved only by improving their knowledge and beliefs [23].

There is no significant difference in the adherence status of studied students in relation to their socio-demographic variables (age, residence, and family income) and the academic variables (studying year and academic score). The absence of significance in our study may be due to statistical reasons where a larger sample size was needed to yield significant results. However, it may be due to the effect of the students’ narrow age range (18–26) and their educational similarity which overwhelmed the effect of residence and income differences and led to this insignificant difference. Also, there is no significant difference in relation to their previous infection or infection of their relatives or friends with COVID-19. The only significant difference observed in our study was in relation to the death of a relative or a friend due to COVID-19 where students who have relative or friend deaths were less adherent to the preventive measures. Students and their dead relatives and friends come from the same families and communities which have similar practices. These families and communities were most probably less adherent to COVID-19 precautions and consequently more deaths.

Other studies conducted in Egypt revealed a higher level of adherence to COVID-19 preventive measures in comparison with our study. For example, an Egyptian online study conducted in June 2020 and targeting medical students in Suez Canal University revealed that 92% of students were adherent to COVID-19 preventive measures [24]. Also, similar studies were conducted on university students (medical and nonmedical) in various countries to assess their practice regarding COVID-19 preventive measures. An online cross-sectional KAP study conducted on university students in Japan by Hatabu et al. (2020) revealed that most of the students (96.4%) wore face masks and washed their hands with moderate or high frequency [25]. A study conducted in China by Peng et al. (2020) revealed that 87.94% of students were performing a proactive practice toward COVID-19 [16].

A high level of adherence to COVID-19 preventive measures was also observed in studies that targeted medical students in developing countries like Jordan [20, 21], Iraq [26], Iran [27], Pakistan [28], Afghanistan [29], KSA [30], and UAE [31]. All of these studies revealed highly prevalent good behavior regarding COVID-19 preventive measures among the studied students with very high healthy behaviors scores.

However, a study conducted by Barqawi et al. (2021) in UAE that targeted physicians and medical students revealed that only 18.2% of the studied individuals reported that they would wear the face mask when they came in contact with suspected individuals (having flu-like symptoms) [32].

These different findings between the current study and the other studies with the high level of adherence to COVID-19 preventive measures may be due to the enrollees of these studies where they were both males and females while our study has enrolled only male students who are more likely to be engaged in risky behavior towards COVID-19 than females [16, 18, 33–35]. In addition, most Al-Azhar medical students are from outside Cairo governorate so they live in collective accommodation places as university housing or rented flats. Also, most students have a family income of fewer than 5000 L.E, which obligates the students to depend on public transportation. These factors make social distancing measures difficult to apply. Moreover, legislation and rules that obligate individuals and institutions to be concomitant with COVID-19 preventive measures are strictly applied in some of these countries like Saudi Arabia and China.

However, the most important factor, from our point of view, responsible for this difference between this study and other Egyptian and international studies is the time of the data collection in relation to the emergence of the pandemic. Almost all these studies were conducted a short period after disease emergence and even during the lockdown period, which has a role in the students’ behavior, especially items related to self-isolation and social distancing measures [31]. Also, with the predominance of a panic state all over the world at this time, students’ practices may have been affected. On the other side, our study was conducted in March and April 2021, after more than a year had passed since the emergence of the disease in December 2019, where people and countries became more adapted to the existence of disease and more reluctant to apply preventive measures.

### Study limitations and strengths

Selection bias cannot be excluded due to lack of randomization in the participants’ selection. These facts make the generalization of our findings problematic.

Although our study was restricted to a certain age, sex, and educational level, this category represents a considerable proportion of the Egyptian population and their role as a viral transmitter to other vulnerable groups cannot be neglected. However, this study has a privilege compared with other studies as it was conducted after the lapse of adequate time since the start of the pandemic, and therefore, it depicts the established rather than the emerged norms of practice among medical students.

## Conclusions

Unexpectedly, there is a low level of adherence to COVID-19 preventive measures among male medical students who are assumed to have good knowledge and practices. Although the students’ beliefs toward COVID-19 infection and its preventive measures appear to be the most probable cause, further studies are needed to affirm or exclude this assumption. Also, this study may draw the attention of policymakers to the urgent need for a health education campaign targeting this age category with great emphasis on their role and responsibility towards other vulnerable groups and towards their nation. Furthermore, this study provides information to policymakers about COVID-19 incidence among students (18.8%) and disease and deaths among their relatives and friends. However, these must be taken with caution because we relied only on the students’ reports without any additional confirmatory tools.

## Data Availability

The datasets used and/or analyzed during the current study are available from the corresponding author on reasonable request.

## Abbreviations

COVID-19: Coronavirus disease 2019
UAE: United Arab Emirates
WHO: World Health Organization

## References

[1] Cui J, Li F, Shi ZL (2019). Origin and evolution of pathogenic coronaviruses. Nat Rev Microbiol.

[2] Lai CC, Shih TP, Ko WC, Tang HJ, Hsueh PR (2020). Severe acute respiratory syndrome coronavirus 2 (SARS-CoV-2) and coronavirus disease-2019 (COVID-19): the epidemic and the challenges. Int J Antimicrob Agents..

[3] World Health Organization. Laboratory testing for coronavirus disease 2019 (COVID-19) in suspected human cases: interim guidance, 2020. https://apps.who.int/iris/bitstream/handle/10665/331329/WHO-COVID-19-laboratory-2020.4-eng.pdf?sequence=1&isAllowed=y. Accessed 24 Apr 2021.

[4] World Health Organization. Statement on the second meeting of the International Health Regulations Emergency Committee regarding the outbreak of novel coronavirus (2019-nCoV). Geneva, Switzerland; 2020. https://www.who.int/news/item/30-01-2020-statement-on-the-second-meeting-of-the-international-health-regulations-(2005)-emergency-committee-regarding-the-outbreak-of-novel-coronavirus-(2019-ncov). [Accessed 29 Apr 2021]

[5] Lakshmi PS, Suresh M (2020). Factors influencing the epidemiological characteristics of pandemic COVID-19: a TISM approach. Int J Healthc Manag.

[6] Kampf G, Todt D, Pfaender S, Steinmann E (2020). Persistence of coronaviruses on inanimate surfaces and their inactivation with biocidal agents. J Hosp Infect.

[7] European Center for Disease Prevention and Control. Rapid risk assessment. Coronavirus disease 2019 (COVID-19) in the EU/EEA and the UK – eighth update, 2020. https://www.ecdc.europa.eu/sites/default/files/documents/covid-19-rapid-risk-assessment-coronavirus-disease-2019-eighth-update-8-april-2020.pdf. [Accessed 2 May 2021].

[8] Centers for Disease Control and Prevention (CDC): Scientific Brief: SARS-CoV-2 Transmission. 2021. https://www.cdc.gov/coronavirus/2019-ncov/science/science-briefs/sars-cov-2-transmission.htm. [Accessed at 6 Oct 2021]. 34009775

[9] World Health Organization. Coronavirus disease 2019 (COVID-19) situation report – 26. 2020. Available from: https://www.who.int/docs/default-source/coronaviruse/situation-reports/20200215-sitrep-26-covid-19.pdf?sfvrsn=a4cc6787_2. [Accessed 6 May 2021].

[10] Wikipedia: COVID-19 pandemic in Egypt. https://en.wikipedia.org/wiki/COVID-19_pandemic_in_Egypt. [Accessed 2 May 2021].

[11] World Health Organization. COVID-19 country profile; Egypt. Available at: https://covid19.who.int/region/emro/country/eg. [Accessed 5 May 2021].

[12] Egypt shuts down airports, suspends air travel: PM. (2020). Available at: https://egyptianstreets.com/2020/03/16/egypt-shuts-down-airports-suspends-air-travel-pm/. Accessed 2 May 2021.

[13] BBC News. [Corona virus: one death, 46 new infections in Egypt, and increasing infections in Arab countries] (in Arabic): BBC Arabic, London, England. Available at: https://www.bbc.com/arabic/middleeast-51963726. Accessed 13 May 2021.

[14] El-Sheikh S. Daily news Egypt: what has happened in Egypt since discovering 1st COVID-19 case? Egypt has taken several measures on economic, medical, social, educational levels. 2020. https://wwww.dailynewssegypt.com/2020/03/24/what-has-happened-in-egypt-since-discovering-1st-covid-19-case. [Accessed 28 Apr 2021].

[15] United Nations Egypt: COVID-19. Using new technologies to engage young people and fight misinformation in Egypt. 2020. https://egypt.un.org/en/51263-covid19-using-new-technologies-engage-young-people-and-fight-misinformation-egypt. [Accessd 11 May 2021].

[16] Peng Y, Pei C, Zheng Y, Wang J, Zhang K, Zheng Z, et al. A cross-sectional survey of knowledge, attitude and practice associated with COVID-19 among undergraduate students in China. BMC Public Health. 2020;20(1):1–8. 10.1186/s12889-020-09392-z. 10.1186/s12889-020-09392-z PMC7447607 32847554

[17] Kim JS, Choi JS (2016). Middle East Respiratory Syndrome–related knowledge, preventive behaviours and risk perception among nursing students during outbreak. J Clin Nurs.

[18] Kasemy ZA, Bahbah WA, Zewain SK, Haggag MG, Alkalash SH, Zahran E, et al. Knowledge, attitude and practice toward COVID-19 among Egyptians. J Epidemiol Glob Health. 2020;10(4):378–89. 10.2991/jegh.k.200909.001. 10.2991/jegh.k.200909.001 PMC7758851 33009730

[19] Almutiri TM, Alzhrani WH, Alraddadi R (2020). Adherence to COVID-19 preventive measures and its predictors among the population of Jeddah city 2020. Int J Med Dev Countries..

[20] Khasawneh AI, Humeidan AA, Alsulaiman JW, Bloukh S, Ramadan M, Al-Shatanawi TN, et al. Medical students and COVID-19: knowledge, attitudes, and precautionary measures: a descriptive study from Jordan. Front Public Health. 2020;8(3):253–69. 10.3389/fpubh.2020.00253. 10.3389/fpubh.2020.00253 PMC7274076 32574313

[21] Alzoubi H, Alnawaiseh N, Al-Mnayyis A, Lubad MA, Aqel A, Al-Shagahin H (2020). COVID-19-knowledge, attitude and practice among medical and non-medical university students in Jordan. J Pure Appl Microbiol..

[22] Lee M, Kang BA, You M (2021). Knowledge, attitudes, and practices (KAP) toward COVID-19: a cross-sectional study in South Korea. BMC Public Health..

[23] Moradzadeh R, Nazari J, Shamsi M, Amini S (2020). Knowledge, attitudes, and practices toward coronavirus disease 2019 in the central area of Iran: a population-based study. Front Public Health..

[24] Soltan EM, El-Zoghby SM, Salama HM (2020). Knowledge, risk perception, and preventive behaviors related to COVID-19 pandemic among undergraduate medical students in Egypt. SN Compr Clin Med..

[25] Hatabu A, Mao X, Zhou Y, Kawashita N, Wen Z, Ueda M, et al. Knowledge, attitudes, and practices toward COVID-19 among university students in Japan and associated factors: an online cross-sectional survey. PLoS One. 2020;15(12):e0244350. 10.1371/journal.pone.0244350. 10.1371/journal.pone.0244350 PMC7751858 33347488

[26] Khalil NS, Al-Yuzbaki DB, Tawfeeq RS (2020). COVID-19 knowledge, attitude and practice among medical undergraduate students in Baghdad city. Eurasian J Biosci..

[27] Taghrir MH, Borazjani R, Shiraly R (2020). COVID-19 and Iranian medical students; a survey on their related-knowledge, preventive behaviors and risk perception. Arch Iran Med..

[28] Noreen K, Rubab ZE, Umar M, Rehman R, Baig M, Baig F (2020). Knowledge, attitudes, and practices against the growing threat of COVID-19 among medical students of Pakistan. PloS One..

[29] Nemat A, Raufi N, Sediqi MF, Rasib AR, Asady A (2021). Knowledge, attitudes, and practices of medical students regarding COVID-19 in Afghanistan: a cross-sectional study. Risk Manag Healthc Policy..

[30] Alrasheedy AA, Abdulsalim S, Farooqui M, Alsahali S, Godman B (2021). Knowledge, attitude and practice about Coronavirus disease (COVID-19) pandemic and its psychological impact on students and their studies: a cross-sectional study among pharmacy students in Saudi Arabia. Risk Manag Healthc Policy..

[31] Baniyas N, Sheek-Hussein M, Al Kaabi N, Al Shamsi M, Al Neyadi M, Al Khoori R (2021). COVID-19 knowledge, attitudes, and practices of United Arab Emirates medical and health sciences students: a cross sectional study. PloS One..

[32] Barqawi HJ, Kampani DD, Haddad ES, Al-Roub NM, Abu-Gharbieh E (2021). Readiness of physicians and medical students to cope with the COVID-19 pandemic in the UAE. PloS One..

[33] Qutob N, Awartani F (2021). Knowledge, attitudes and practices (KAP) towards COVID-19 among Palestinians during the COVID-19 outbreak: a cross-sectional survey. PloS One..

[34] Al-Hanawi MK, Angawi K, Alshareef N, Qattan AM, Helmy HZ, Abudawood Y (2020). Knowledge, attitude and practice toward COVID-19 among the public in the Kingdom of Saudi Arabia: a cross-sectional study. Front Public Health..

[35] Ali RA, Ghaleb AA, Abokresha SA (2021). COVID-19 related knowledge and practice and barriers that hinder adherence to preventive measures among the Egyptian community: an epidemiological study in Upper Egypt. J Public Health Res..

